# Isolation and Characterization of cDNAs Encoding Leucoanthocyanidin Reductase and Anthocyanidin Reductase from *Populus trichocarpa*

**DOI:** 10.1371/journal.pone.0064664

**Published:** 2013-05-31

**Authors:** Lijun Wang, Yuanzhong Jiang, Li Yuan, Wanxiang Lu, Li Yang, Abdul Karim, Keming Luo

**Affiliations:** 1 Key Laboratory of Eco-environments of Three Gorges Reservoir Region, Ministry of Education, Institute of Resources Botany, School of Life Sciences, Southwest University, Chongqing, China; 2 College of Horticulture and Landscape Architecture, Southwest University, Chongqing, China; 3 Key Laboratory of Adaptation and Evolution of Plateau Biota, Northwest Institute of Plateau Biology, Chinese Academy of Sciences, Xining, China; Northwestern University Feinberg School of Medicine, United States of America

## Abstract

Proanthocyanidins (PAs) contribute to poplar defense mechanisms against biotic and abiotic stresses. Transcripts of PA biosynthetic genes accumulated rapidly in response to infection by the fungus *Marssonina brunnea* f.sp. *multigermtubi*, treatments of salicylic acid (SA) and wounding, resulting in PA accumulation in poplar leaves. Anthocyanidin reductase (ANR) and leucoanthocyanidin reductase (LAR) are two key enzymes of the PA biosynthesis that produce the main subunits: (+)-catechin and (−)-epicatechin required for formation of PA polymers. In *Populus*, *ANR* and *LAR* are encoded by at least two and three highly related genes, respectively. In this study, we isolated and functionally characterized genes *PtrANR1* and *PtrLAR1* from *P. trichocarpa*. Phylogenetic analysis shows that *Populus ANR1* and *LAR1* occurr in two distinct phylogenetic lineages, but both genes have little difference in their tissue distribution, preferentially expressed in roots. Overexpression of *PtrANR1* in poplar resulted in a significant increase in PA levels but no impact on catechin levels. Antisense down-regulation of *PtrANR1* showed reduced PA accumulation in transgenic lines, but increased levels of anthocyanin content. Ectopic expression of *PtrLAR1* in poplar positively regulated the biosynthesis of PAs, whereas the accumulation of anthocyanin and flavonol was significantly reduced (*P*<0.05) in all transgenic plants compared to the control plants. These results suggest that both *PtrANR1* and *PtrLAR1* contribute to PA biosynthesis in *Populus*.

## Introduction

Flavonoids are a large group of plant secondary metabolites that comprise several classes of compounds (e.g. anthocyanins, flavonols, isoflavones and flavan-3-ols) and accumulate in a wide variety of plant tissues [1]. Proanthocyanidins (PAs), also called as condensed tannins, are oligomers or polymers of flavan-3-ols and are among the major flavonoid compounds found in higher plants [2], [3]. As a final product of the flavonoid pathway, PAs play an important role in the protection of plants against herbivores and pathogens [1], [4], [5]. PAs in forage crops can protect ruminants against pasture bloat [6], [7], [8]. PAs also act as antioxidants with beneficial effects for human health by protection against free radical-mediated injury and cardiovascular disease [9], [10]. Additionally, PAs also contribute to the astringency and taste of many fruits and the quality of other plant products, such as wine, tea (*Camellia sinensis*), and cocoa [11]. Therefore, an understanding of the mechanisms leading to the formation of PA polymers and its regulation is important for regulation of PA biosynthesis in plants.

Flavonoids are synthesized through the phenylpropanoid pathway. PA biosynthesis shares the common ﬂavonoid biosynthetic pathway with anthocyanins and ﬂavonols. The common pathway has been characterized genetically and biochemically in a number of plant species including *Arabidopsis thaliana* and *Medicago truncatula* [12]. At the beginning of the flavonoid biosynthetic pathway, the enzyme chalcone synthase (CHS) carries out the condensation of one molecule of 4-coumaroyl-CoA and three molecules of malonyl-CoA to yield naringenin chalcone. Chalcone is isomerised to a flavanone via the enzyme chalcone flavanone isomerase (CHI). Sharing these central intermediates, the pathway divided into several side branches, each producing a different class of flavonoids. The stereospecific 3ß-hydroxylation of (2S)-flavanones are catalyzed to dihydroflavonols by flavanone 3-hydroxylase (F3′H). For the biosynthesis of anthocyanins, dihydroflavonols are catalyzed to flavan-3,4-diols (leucoanthocyanins) by dihydroflavonol reductase (DFR), then leucoanthocyanins are converted to anthocyanidins by anthocyanidin synthase (ANS). The formation of glucosides is catalyzed by UDP glucose-flavonoid 3-o-glucosyl transferase (UFGT), which stabilizes the anthocyanidins by 3-O-glucosylation [13], [14].

The biosynthesis of PAs and anthocyanins begins with the same upstream ﬂavonoid pathway, leading to the synthesis of ﬂavan-3-ol units such as catechin and epicatechin [15], [16]. Catechin is traditionally derived from leucocyanidin by the catalyzation of leucoanthocyanidin reductase (LAR) [15], while epicatechins are synthesized from cyanidin by anthocyanidin reductase (ANR), which is encoded by the BANYULS (*BAN*) gene and has been initially characterized in *Arabidopsis* [17]. Ectopic expression of *BAN* in tobacco flower petals and *Arabidopsis* leaves results in loss of anthocyanins and accumulation of PAs, suggesting that there is an interaction between anthocyanidin and PA pathways [17]. *VvANR*, encoded by a single gene, has been also isolated and characterized in grapevines (*Vitis vinifera*) [18]. More recently, three *ANR* genes were reported in apple (*Malus*×*domestica* Borkh.), and introduction of these genes into tobacco inhibits expression of both *CHI* and *DFR* genes in flowers, leading to loss of anthocyanin [19]. This finding suggests that the *ANR* gene may be capable of generating catechin *via* an alternative route.

LAR activity has been found in several plants and its activity correlated with PA accumulation [2], [15], [20], [21]. The functionality of LAR has been determined in *Desmodium uncinatum*, in which the recombinant LAR protein catalyzed the conversion of leucocyanidin, leucodelphinidin, or leucopelargonidin to the corresponding 2,3-trans-ﬂavan-3-ol (catechin) [5]. This finding clearly established the role of DuLARs in PA biosynthesis. Two grapevine *LAR* orthologs were also isolated and had different patterns of expression in skin and seeds [18]. In *M. truncatula*, a single *LAR* gene has been cloned and characterized and transgenic tobacco plants constitutively overexpressing *MtLAR* showed reduced anthocyanin content, but no catechin or increased levels of PAs were detected either in leaves or in flowers, suggesting the poor correlation between *LAR* expression and PA biosynthesis in *Medicago* [22]. In addition, two *LAR* genes were reported in *Lotus corniculatus*, but only *LcLAR1* produced active proteins following heterologous expression in *Escherichia coli* [21]. Therefore, these studies mentioned above suggest a complex genetic control for PA biosynthesis in the same plant species.

Poplar (*Populus* spp.) is an important forest tree with significant economic and ecological application, but many native poplar species are susceptible to diseases caused by *Melampsora medusae, Marssonina brunnea* and *Septoria musiva*. The flavonoid-derived PAs are one class of the major defense phenolics in poplar. Therefore, it is important to develop a model system for studies on the biochemical and genetic mechanisms of PA biosynthesis in tree and woody perennial plants [23]. The transcriptional response of hybrid poplar (*P. trichocarpa* × *P. deltoides*) to poplar leaf rust (*M. medusae*) infection showed that these genes for enzymes of PA biosynthesis were strongly induced in the infection process, linking this pathway for the first time to the pathogen defense response in poplar [24]. Similarly, these *M. medusae*–induced gene family members of PA biosynthesis pathway correspond to those genes most highly induced by wounding in leaves of *P. fremontii* ×*P. angustifolia* [25]. With the completion of the *P. trichocarpa* genome sequence, bioinformatic analysis showed that three putative *LAR* genes (*PtrLAR1*, *PtrLAR2* and *PtrLAR3*) were identified in *Populus* genome [25]. When displayed in a phylogenetic tree, PtrLAR1/PtrLAR2 and PtrLAR3 proteins occurred in two distinct phylogenetic lineages [25]. We have demonstrated previously that a *Populus LAR*, *PtrLAR3*, was expressed in various tissues and the highest level expression in roots. Overexpression of *PtrLAR3* in Chinese white poplar (*P. tomentosa* Carr.) led to a significant increase in PA levels and improved resistance to fungal pathogens in transgenic plants [26]. However, it is not yet clear whether PtrLAR1 is involved in PA biosynthesis in *Populus*.

In this report, transcription profiles of flavonoid genes of poplar leaves in a compatible interaction with wounding, salicylic acid (SA) and fungal pathogens were analyzed by *quantitative RT-PCR*. Furthermore, these genes encoding ANR and LAR isoforms from *P. trichocarpa* were cloned and characterized. Expression analysis of these genes in the empty-vector control and transgenic lines showing different accumulation of PAs has developed our understanding of the genetic control of PA biosynthesis in poplar. Our findings established the roles of PtrANR and PtrLAR in the PA biosynthesis pathway in poplar and may be of importance in understanding the genetic mechanisms controlling PA accumulation in poplar as well as other plants.

## Materials and Methods

### Plant Growth Conditions and Stress Treatments

*Populus tomentosa* Carr. (clone 73) is native in China. In this study, we collected poplar leaves from the vicinity of Yangling. No specific permission was required for any locations/activities. It was sure that the field studies did not involve endangered or protected species. Poplar plants were grown in the greenhouse at 25°C under a 14-/10-h light/dark cycle with supplemental light (4500 lux). Three-month-old poplar seedlings grown in the greenhouse were subjected to stress experiments analysis of transgenics. Upon harvest of leaves, midveins and necrotic tissue were removed and tissues were frozen in liquid nitrogen and stored at −80°C until analyzed. Leaves within the range of Leaf Plastochron Index (LPI)-8 to −12 [27], which correspond to the youngest fully expand leaves, were used for all stress experiments.

For pathogen inoculations, leaf discs were inoculated with *Melampsora brunnea* f.sp. *multigermtubi* (*Mb*) as previously described [26]. Briefly, leaf discs were inoculated with *Mbm* urediniospores suspended in 0.01% Tween 20 at a density of 800–1000 spores cm^−2^. Control samples were mock-inoculated with 0.01% Tween 20. After inoculation, leaves were kept on wet paper in Petri dishes and incubated in a growth chamber at 18°C with a 16-h photoperiod. Infected and control leaves were harvested at 3 days post inoculation. For salicylic acid (SA) treatment, solution with 5 mM was generously sprayed on leaves until saturation. The Leaves were harvested 24 h after treatments. The wounding treatment consisted of making small holes on leaves with disposable syringe. Control plants were untouched for the wound treatment or sprayed with H_2_O for elicitor treatment. All tested tissues were harvested and frozen in liquid nitrogen until further processing. Biological materials were collected from three replicate trees and processed separately for each treatment.

Plant height from the base of the stem to leaf plastochron index (LPI)-0, of the youngest unfurled leaf, 3 cm in length [27], was recorded, and the petiole of LPI-0 was marked. LPI-1 to LPI-5 of three plants for each genotype were monitored every third day over a 9-day period for the analysis.

### Cloning of *PtrLAR1* and *PtrANR1*

Total RNA was isolated from frozen tissues of poplar plants using a RNA RNeasy Plant Mini Kit (Qiagen, Germany) following the manufacturer’s instructions. Leaves and petioles were excised from stems, including the fourth (young) and fifth (mature) internodes from the top of the stems. First-strand cDNA was synthesized from 2 µg DNase-treated RNA with RT-AMV transcriptase (TaKaRa, Dalian, China) in a total volume of 20 µl using oligo d(T) at 42°C for 30 min. The full open reading frame of *PtrLAR1* was amplified with gene-specific primers (LAR1-F: 5′-GCATGACTGTTTCAGCTTCT-3′; LAR1-R: 5′-TCACACCAACAAACCACA GG-3′) Joint Genome Institute, http://genome.jgi-psf.org/poplar/poplar.info.html) by RT-PCR with cDNA from roots. The PCR reaction was carried out with Pfu DNA polymerase (TaKaRa) in a total volume of 50 µl with an initial denaturing step at 94°C for 3 min, 32 cycles of 94°C for 45 s, 56°C for 45 s, and 72°C for 90 s and a final extension step at 72°C for 10 min. The amplification products were cloned into the plant binary vector pCXSN, which is a zero-background TA cloning system that provides simple and high-efficiency direct cloning of PCR-amplified DNA fragments [28]. A 1155-bp PCR-fragment of PtrANR1 was amplified from poplar cDNA with gene-specific primers (ANR1-F:5′-GCTAGA**AAGCTT**GCATAGCATCCCAGTTGACC-3′; ANR1-R: 5′-GTAGC**TCTAGA**TGACGCTAGATTGCTTCAGC-3′). Restriction enzyme sites *Hin*dIII (in the ANR1-F primer) and *Xba*I (in the ANR1-R primer) were marked in bold, which were used for subcloning. After verification by DNA sequence analysis in both directions, the fragment digested with *Hin*dIII/*Xba*I was inserted into the corresponding sites of pBI121 (Clontech). The resulting vectors *35S:PtrLAR1* and *35S:PtrANR1*, contain the open reading frame down-stream of the cauliflower mosaic virus 35S promoter and the hygromycin phosphotransferase gene (*Hpt*) as well as neomycin phosphotransferase II (*NPTII*) as a plant-selectable marker conferring hygromycin and kanamycin resistance, respectively. These plant transformation vectors were transferred into *A. tumefaciens* EHA105 by the freeze–thaw method [29].

### Transformation of *P. tomentosa* Carr. Plants

Transgenic Chinese white poplar (*P. tomentosa* Carr.) plants were generated by *Agrobacterium*-mediated transformation as described previously [30]. Recombinant *Agrobacterium* was used to infect poplar leaf discs and putative transgenic plants were selected on woody plant medium (WPM) [31] supplemented with 10 mg l^-1^ hygromycin or 25 mg l^−1^ kanamycin. Rooted plantlets were acclimatized in pots at 25°C in a 14/10 light/dark cycle and then transferred to the greenhouse for further studies.

### DNA Extraction and PCR Analysis

Genomic DNA was extracted from leaves (300 mg) of untransformed control and hygromcyin-resistant and kanamycin-resistant plants using the modified cetyltrimethylammonium bromide extraction method as previously described [30]. To determine the presence of transgenes, putative transgenic plants were screened preliminarily by PCR analysis [32]. The following primers were designed for *Hpt* and *NPTII*: Hpt-F: 5′-ATCGGACGATTGCGTCGTCGCATC-3′, Hpt-R: 5′-GTGTCACGTTGCAAGA CCTG-3′; NPTII-F:5′-AGGCTATTCGGCTATGACTGG-3′, NPTII-R:5′-GCCATGGGTCACGACGAGATC-3′. The PCR conditions were an initial denaturing step at 94°C for 3 min and 35 cycles of 94°C for 30 s, 60°C (*Hpt*), 56°C (*NPTII*) for 30 s, and 72°C for 1 min. The amplification products were resolved on a 1% (w/v) agarose gel and visualized after ethidium bromide staining.

### Semi-quantitative RT-PCR and Quantitative Real-time PCR Analysis

Total RNA was extracted from leaves, roots, stems, and petioles of poplar plants and treated with DNase I (TaKaRa) according to the manufacturer’s instructions. All RNA was purified and first-strand cDNA was synthesized as described above. The reverse-transcribed cDNA samples were used for quantitative real-time PCR, which was performed on a TaKaRa real-time-PCR detection system. 18S rRNA was used as an internal control. The RT-PCR conditions were an initial denaturation step at 94°C for 3 min, 28 cycles of 94°C for 30 s, 58°C (*PtrLAR1*), 55°C (*PtrANR1*) for 30 s, and 72°C for 1 min, and an extension step at 72°C for 10 min. The amplification products were resolved by 1% (w/v) agarose gel electrophoresis and visualized with ethidium bromide under UV light. Quantitative real-time PCR analysis was performed as described Tsai et al. [25] in a 20 µl reaction volume containing 10 µl of SYBR Green master mix reagent (TaKaRa). The primers were designed using Primer 5.0 software: forward and reverse primers for *PtrLAR1* and *PtrANR1* amplifications were PtrLAR1-F (5′-ACCTACTTGCTATTGCTGCAG-3′), PtrLAR1-R(5′-CCAAGGTA CGAAAAGCTTCAT-3′), PtrANR-F(5′-ACAGGGTTTGTGGCATC-3′) and PtrANR-R (5′-TCTGGGAGCATTGAAGC-3′). Each reaction was performed in duplicate and with three biological replicates along with no-template controls. The gene-quantification method was based on the relative expression of the target gene versus the reference gene (*18S*) [25].

### Phylogenetic Analysis

The deduced amino acid sequences were analysed using the program DNAMAN and the software MAGE version 4.0 (Lynnonon Biosoft, Quebec, Canada). Alignment of the deduced amino acid sequences was performed using DNAMAN. The phylogenetic relationships of LARs were analyzed with the neighbour-joining method using MAGE version 4.0.

### Extraction and Quantification of Proanthocyanidins, Anthocyanins and Flavonols

For extraction of PAs, tissues were ground in liquid nitrogen and 500 mg of ground tissue was used for extraction in 5 ml extraction buffer (70% [V/V] acetone containing 0.1% [W/V] ascorbic acid) for 24 h at room temperature on a rotating shaker in darkness. The water phase was separated from the acetone phase by adding sodium chlorid etosaturation. After removal of the acetone phase, the water phase was extracted with additional sodium chloride-saturated 100% acetone, and the resulting acetone phase was combined with the first acetone phase. The samples were dried under a stream of nitrogen, the pellet re-dissolved in 2 ml of methanol acidified with 0.1% ascorbic acid, centrifuged at 16,100 g for 10 min, and the final supernatant kept in darkness and under refrigeration until analysis.

The insoluble PA content was tested using the butanol/HCl method [33]. The residues from the above tissue extractions were dried in air for 2 days, and then 1 ml butanol/HCl reagent was added and the mixture was sonicated at room temperature for 60 min and centrifuged at 2,500 g for 10 min. Supernatants were transferred to cuvettes for determination of absorption at 550 nm and were then boiled for 1 h. After cooling to room temperature, the A_550_ was recorded again and the first value subtracted from the second. Absorbance values were converted into PA equivalents using a standard curve (2.5, 5, 10, 20, and 40 mg) of procyanidin B1 (Indofine). Three independent experiments were performed for each sample.

For analysis of anthocyanin levels, 5 ml of methanol:0.1% HCl was added to 0.5 g of ground tissue and sonicated for 1 h, followed by shaking overnight at 120 rpm. After centrifugation at 2,500 g for 10 min, 1 ml of water was added to 1 ml of extract, followed by addition of 1 ml of chloroform to remove chlorophyll. Absorption of the clear supernatant was then measured at 530 nm. Total anthocyanin concentration was calculated using the molar absorbance of cyanidin-3-O-glucoside.

For analysis of flavonols, 100 mg of plant tissue was extracted with 3 ml 80% methanol, sonicated for 1 h, and allowed to stand overnight at 4°C. The extract was centrifuged to remove debris and the supernatant dried under nitrogen. Dried samples were incubated with 3 ml of 1 N HCl at 90°C for 2 h and extracted twice with 3 ml of ethyl acetate. Ethyl acetate extracts were pooled, dried under nitrogen, and resuspended in 200 µl of methanol.

HPLC analysis and quantification of total anthocyanins and PA-derived compounds harvested from transgenic and control plants were performed using a LC-20AD HPLC system (Shimadzu Co., Kyoto, Japan) as described in Deluc *et al*. [34]. Separation was performed with a linear elution gradient from A (0.2% phosphoric acid in H2O) and B (acetonitrile) over 30 min at a flow rate of 0.5 ml min^−1^. For favonols separation, 60% methanol solution was used for isocratic eluting with volume of injecting sample of 10 µl and at a flow-rate of 1 ml min^-1^. In each case, the column was maintained at 40°C and the diode array detector was used to record absorption at 280 nm and 368 nm. Catechin, epicatechin, kaempferol and quercetin were used to create standard absorption curves.

### Histochemical Staining with DMACA

DMACA reacts quite specifically with flavan 3-ol monomers and PAs to form a blue chromophore [35]. Histochemical analysis of PA accumulation in various tissues was detected as described by Li *et al*. [36]. In brief, plant tissues were decolourized in 5 ml of 30% acetic acid in ethanol for 12 h and washed with 75% ethanol. PAs were detected by staining tissues for 3 h with 1% (w/v) DMACA in ethanol: 6 N HCl (1∶1, v/v). Images of stem and petiole sections were recorded using a Nikon microscope. Figures were formatted and assembled with Adobe Photoshop 7.0.

## Results

### Stress-induced Expression of PA Biosynthetic Genes in Poplar

**Figure 1:**
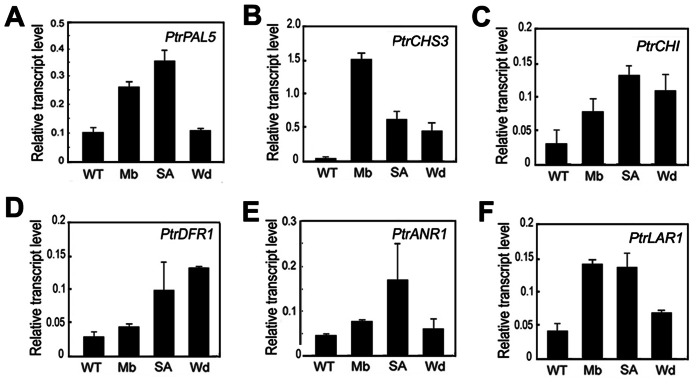
Expression analysis of flavonoid biosynthesis genes in response to different stresses in poplar. For stress treatments, the wild-type plants inoculated with *Marssonina brunnea f.sp. multigermtubi* (Mb), sprayed with salicylic acid (SA),or pricked for wound treatment (Wd). WT indicates the control. Expression was determined by real-time RT-PCR analysis using *18S* as the housekeeping gene. The genes used for analysis are indicated. *PAL* phenylalanine ammonia lyase, *CHS* chalcone synthase, *CHI* chalcone isomerase, *DFR* dihydroflavonol 4-reductase, *ANR* anthocyanidin reductase, *LAR* leucoanthocyanidin reductase. Each column represents the mean value of three independent experiments with error bars indicating ± SD.

PA biosynthesis is often induced by various stresses such as mechanical wounding, pathogen infection and insect herbivory in poplar [4], [24], [37]. In our previous study, transcriptional profiling of PA biosynthetic genes was detected following infection by the fungus *Marssonina brunnea* f.sp. *multigermtubi* using digital gene expression (DGE) analysis. The result demonstrated that at least one gene in each enzymic step was upregulated, with the exception of cinnamate 4-hydroxylase (C4H), resulting in PA accumulation in leaves [26]. To investigate association of the *Populus* flavonoid genes with PA biosynthesis, we carried out gene-specific quantitative real-time polymerase chain reaction (qPCR) analysis using a set of induced flavonoid pathway genes and noninduced genes for comparison. The result showed that transcript abundance of these flavonoid genes essential for PA biosynthesis was induced by fungal infection, treatments of SA and wounding (Fig. 1). The qPCR expression profiles confirmed the trends seen on the DGE analysis for most of PA biosynthesis-related genes.

### Isolation and Characterization of *PtrLAR1* and *PtrANR1* from *P. trichocarpa*

With the completion of the *P. trichocarpa* genome sequence, all enzymatic steps required for PA biosynthesis annotated in the poplar genome [25], [38]. LAR catalyzed early steps in PA biosynthesis are NAPDH-dependent reductases, encoded by three genes in *Populus* [25].

All three *PtrLAR* genes are composed of five exons and four introns (Fig. S1A). PtrLAR1/PtrLAR2 and PtrLAR3 proteins occurred in two distinct lineages in a phylogenetic tree [25]. The *PtrLAR3* gene was previously isolated and overexpression of *PtrLAR3* in Chinese white poplar (*P. tomentosa* Carr.) led to a significant plant-wide increase in PA levels [26]. In the present study, a full-length cDNA sequence of *PtrLAR1* was cloned by reverse transcription (RT)-PCR and contained an open reading frame (ORF) of 1,056 bp encoding a putative protein of 352 amino acids. Sequence comparison of PtrLAR1 showed the highest identity with LAR proteins from *Malus domestica* (69.83%), *Diospyros kaki* (66.85%), *Lotus corniculatus* (62.61%), *L. uliginosus* (62.61%), *Medicago truncatula* (57.91%) and *Desmodium uncinatum* (57.70%) (Fig. S1B). The highly conserved putative motifs (RFLP, ICCN and THD) found in other *LAR* genes [5], [18] was also the deduced PtrLAR1 protein.

Two possible ANR sequences were identified based on the sequences deposited in the *Populus* genome database (Phytozome version 2.0). The duplicated ANR genes, named *PtrANR1* and *PtrANR2*, have similar exon/intron structures (Fig. S2A) and exhibited similar expression patterns, both with a high transcript level in roots [25]. The cDNA fragment of *PtrANR1* with homology to the *Arabidopsis* ANR (AtANR) was cloned from RNA isolated from *P. trichocarpa* leaves and contained an ORF of 1,005 bp encoding a protein of 335 amino acids. PtrANR1 has 62.06% identity to ANR from *Arabidopsis*, 76.18% identity to ANR from *D. kaki*, 68.14% identity to ANR from *L. corniculatus*, 77.29% identity to ANR from *L. uliginosus*, 81.42% identity to ANR, 72.86% identity to ANR from *M. truncatula* and 80.77% identity to ANR from *Vitis vinifera* in the coding region (Fig. S2B). Analysis of *PtrANR1* indicated that the highly conserved putative NAPDH-binding domain reported in the other ANR genes [21] was also present (Fig. S2B).

**Figure 2:**
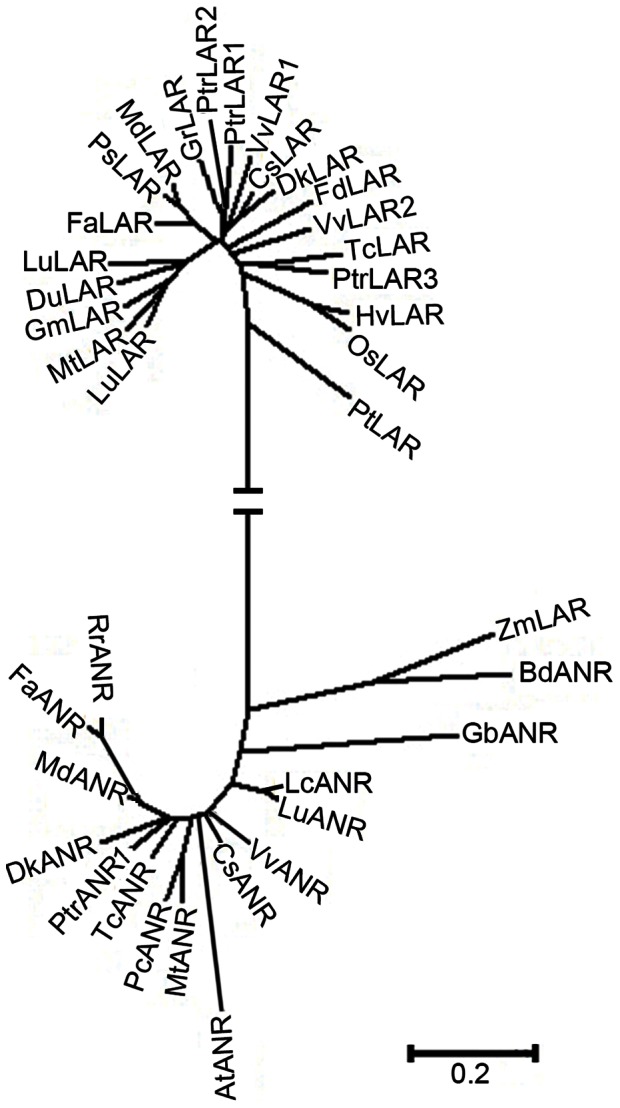
Phylogenetic relationships of LAR and ANR proteins from *P. trichocarpa* Carr. and other plant species. Phylogenetic analyses was performed using the neighbor-joining method by the MEGA version 4 program [45].The scale bar represents 0.2 substitutions per site. GenBank accession numbers are as follows (in parentheses): PtLAR (*Pinus taeda*, CAI56321); OsLAR (*Oryza sativa*, ABF95070); HvLAR (*Hordeum vulgare*, CAI56320); TcLAR (*Theobroma cacao*, ADD51358); FdLAR (*Fagopyrum dibotrys*, AEY62396); DkLAR (*Diospyros kaki*, ACI41981); LcLAR (*Lotus corniculatus*, ABC71329); MtLAR (*Medicago truncatula*, XP_003591830.1); FaLAR (*Fragaria x ananassa*, ABH07785.2); PsLAR (*Pyrus communis*, ABB77697.1); GmLAR (*Glycine max*, AEM23933); GrLAR (*Gossypium arboretum*, CAI56323); VvLAR1 (*Vitis vinifera*, CAI26310); CsLAR (*Camellia sinensis*, ADZ58167); VvLAR2 (*V. vinifera*, CAI26308); LuLAR (*Lotus uliginosus*, AAU45392); MdLAR (*Malus* x *domestica*, AAZ79364); DuLAR (*Desmodium uncinatum*, CAD79341); PtrLAR1 (*P. trichocarpa*, EEE89746); PtrLAR3 (*P. trichocarpa*, EEF06163); RrANR *(Rosa roxburghii*, AFD33553); FaANR (*F. ananassa*, ABD95362); MdANR (*M. domestica*, AEL79861); DkANR (*D. kak*i, BAF56654); TcANR (*T. cacao*, ADD51354); PcANR (*Phaseolus coccineus*, CAD91909); MtANR (*M. truncatula*, AAN77735); CsANR (*C. sinensis*, AAT68773); VvANR (*V. vinifera*, BAD89742); LuANR (*L. uliginosus*, ABM90632); LcANR (*L. corniculatus,* ABC71336); GbANR (*Ginkgo biloba*, AAU95082); BdANR (*Brachypodium distachyon*, XP_003580615); ZmLAR (*Zea mays*, NP_001148881); PtrANR (*P. trichocarpa*, XP_002317270).

A phylogenetic tree was constructed using the predicted amino acid sequences of the putative LAR and ANR proteins from poplar as well as other species. LAR and ANR are related members of the reductase epimerase-dehydrogenase (RED) protein superfamily. As shown in Fig. 2B, PtrLAR1 and PtrLAR2 proteins belonged to one group, while PtrLAR3 was clustered into a distinct branch with TcLAR from *Theobroma cacao.* This finding is consistent with a recently published phylogenetic analysis [26]. The deduced amino acid sequences of PtrANR1 and ANRs from several plant species were also subjected to phylogenetic analysis. The results demonstrated that PtrANR1 was more closely related to ANR proteins than other RED superfamily proteins (Fig. 2), suggesting that PtrANR1 is more likely to have function of anthocyanidin reductase rather than other RED protein functions.

### Expression Profiles of *PtrANR1* and *PtrLAR1,* and PA Accumulation in Poplar Leaves

**Figure 3:**
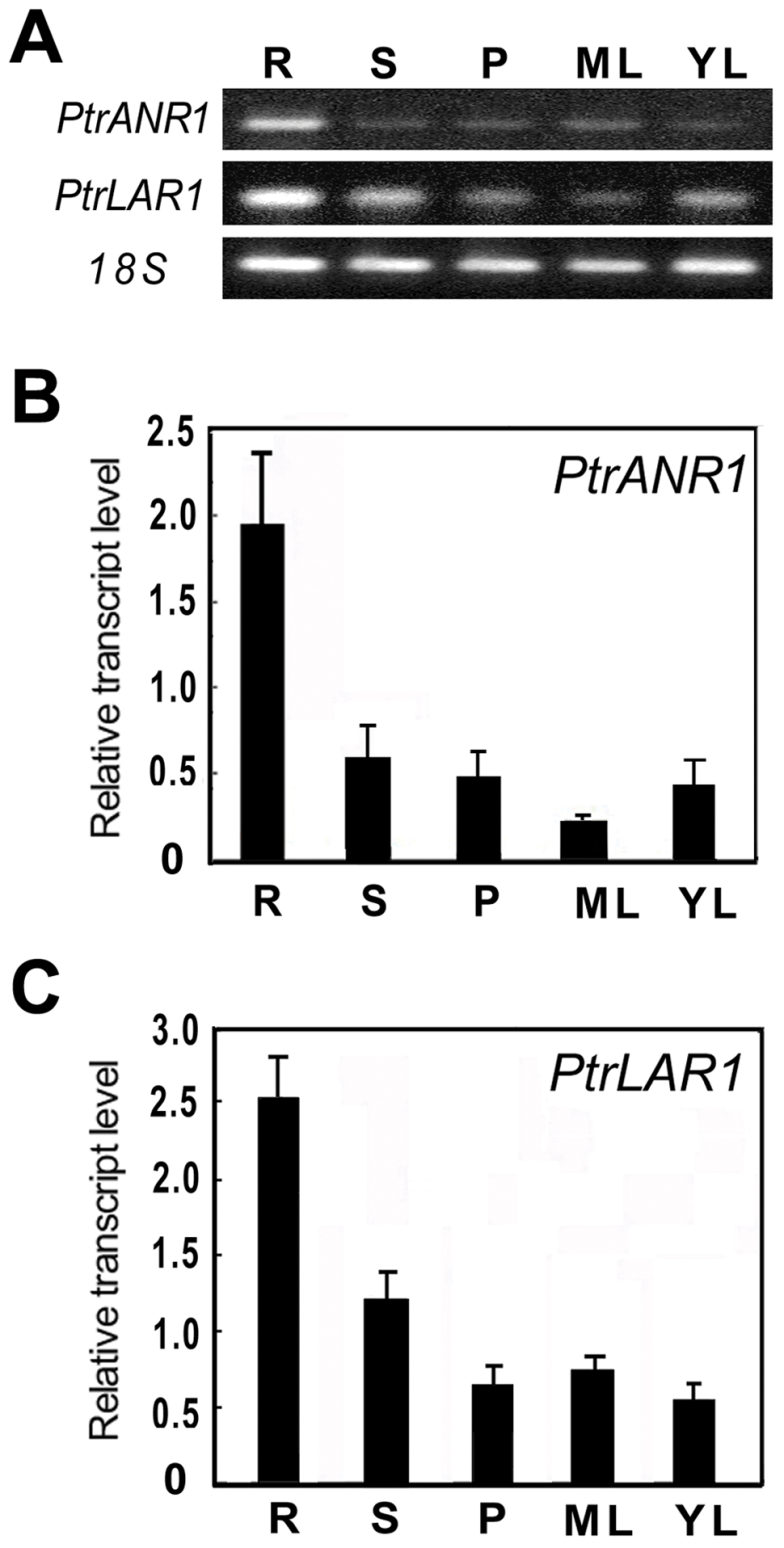
Expression analysis of *PtrANR1* and *PtrLAR1* in *P. trichocarpa* tissues. (A) Semi-quantitative RT-PCR analysis of *PtrANR1* and *PtrLAR1* expression in various tissues of *P. trichocarpa*. (B) Quantitative real-time PCR analysis of *PtrANR1* transcript levels in various tissues of *P. trichocarpa.* (C) Quantitative real-time PCR analysis of *PtrLAR1* transcript levels in various tissues of *P. trichocarpa*. Poplar *18S* expression was used as a control. Total RNA was isolated from roots (R), stems (S), petioles (P), mature leaves (ML), and young leaves (YL).

The expression profiles of *PtrANR1* and *PtrLAR1* were investigated by semi-quantitative RT-PCR with total RNA from various tissues of poplar. Transcript accumulation was detected in all analyzed tissues, including roots, stems, leaves and petioles but the highest mRNA level was found in roots (Fig. 3A), consistent with PA accumulation in different tissues of *P. trichocarpa* [26]. Expression patterns of the *PtrANR1* and *PtrLAR1* genes were further confirmed by quantitative real-time PCR. As shown in Fig. 3B, *PtrANR1* transcripts in the roots were approximately 3-fold higher than in the stems and petioles, and were more than 15 times as abundant as in the mature leaves. Expression of *PtrLAR1* showed a similar pattern to *PtrLAR3* as reported previously by Tsai *et al*. [25], with the highest expression level in roots, with lower expression in other organs (Fig. 3C).

**Figure 4:**
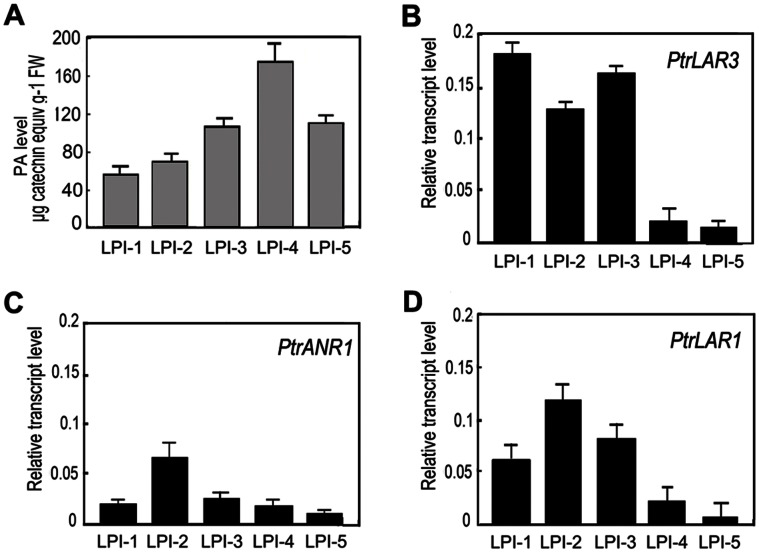
Gene expression of *PtrLAR1*, *PtrLAR3*, *PtrANR1* and accumulation of PAs in wild-type poplar leaves at *different growth stages.* * The leaves of different stages were* harvested from the same plant for every experiment. *The stages correspond to: LPI-1, apex and the second leaves; LPI-2, the fourth leaf; LPI-3, the sixth leaf; LPI-4, the eighth leaf; LPI-5,the tenth leaf*. *(A–C)* Expressions of *PtrLAR1*, *PtrLAR3* and *PtrANR1* in leaves were determined by real-time RT-PCR analysis using *18S* as the housekeeping gene. (D) Quantification of PAs in leaves. All data is presented as mean of three replicates with error bars indicating ± SD.

In a previous study, the relatively high levels of PAs were detected in young leaves of poplar compared with mature leaves [26]. Here, we extracted PAs from developing leaves at various stages. The concentration of PAs was the highest in Stage 4 leaves (around 0.17 mg catechin equivalents/g FW) and was lower in Stage 1 and 2 leaves, indicating that PA synthesis increased in the later stages of leaf development (Fig. 4A). Further, expression patterns for the *Populus* genes encoding LAR and ANR were determined in poplar leaves of different developmental stages. Consistent with their role in PA biosynthesis, *PtrLAR1*, *PtrLAR3* and *PtrANR1* were expressed highly in young leaves (LPI-1, -2 and -3) but there was a sharp decrease in their expression in mature leaves (LPI-4 and -5) (Fig. 4B–C).

### Functional Analysis of *PtrANR1* Gene in Transgenic Poplar Plants

To investigate the function of *PtrANR1*, the ORF in sense or antisense orientation was introduced into *P. tomentosa* Carr. plants for ectopic expression under the control of the cauliflower mosaic virus 35S promoter, respectively. More than twenty kanamycin-resistant lines for each construct were used to produce plants grown to an average height 1.00 m in the greenhouse. PCR analysis using gene-specific primers showed that an expected amplification product specific for *NPTII* was obtained from all transgenic lines tested, whereas no signal was detected from wild-type plants (Fig. S3A), confirming the integration of the transgene into the poplar genome. No phenotypic changes were observed in all of the transgenic lines compared with the controls (Data not shown).

**Figure 5:**
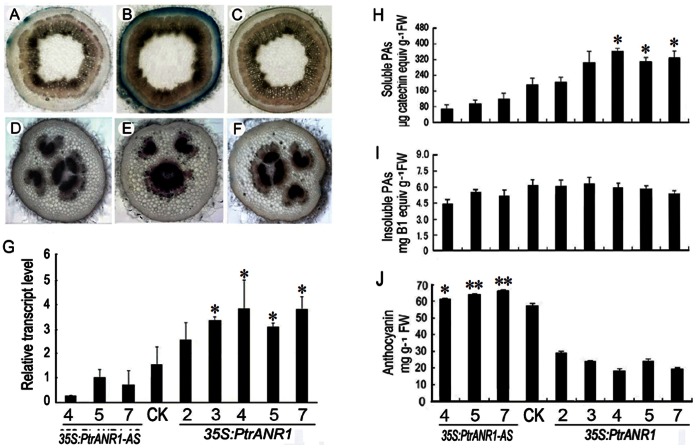
Accumulation of PAs, anthocyanin and gene expression of *PtrANR1* in transgenic *35S:PtrANR1-AS* or *35S:PtrANR1* poplar plants. PAs were localized by staining different tissues of control, *PtrANR1*-overexpressing and *PtrANR1*-antisense (AS) plants with the PA-specific stain dimethylaminocinnamaldehyde (DMACA; blue). (A, D) Control stem, petiole. (B, E) *PtrANR1*-overexpressing stem, petiole. (C, F) *PtrANR1*-antisense stem, petiole. (G) Expression of *PtrANR1* in different transgenic lines was determined by real-time RT-PCR analysis using *18S* as the housekeeping gene. (H) Soluble PA levels in different transgenic lines. (I) Insoluble PA levels in different transgenic lines. (J) Anthocyanin levels as determined by extraction and UV absorption. Numbers refer to independent transgenic lines. CK is empty-vector control line. Asterisks indicate significant differences using Student’s *t*-test (*P*<0.05). All data is presented as mean of three replicates with error bars indicating ± SD.

To estimate PA localization in *PtrANR1*-overexpressing and *PtrANR1-*antisense poplar plants, petiole and stem sections were stained with dimethylaminocinnamldehyde (DMACA), which reacts specifically with PAs and flavan-3-ols to form a blue chromophore [39]. In stem sections, much higher concentration of PAs was observed in the epidermis in the *PtrANR1* overexpressor compared with the control and *PtrANR1-*antisense plants (Fig. 5A–C). In petioles of control plants, DMACA staining showed that PAs were present in the epidermal phloem and xylem cells (Fig. 5D). In *PtrANR1-*antisense petioles, staining was only detected in phloem and xylem layers, while strong staining was observed in these cells of *PtrANR1* overexpressors (Fig. 5E and F).

Spectrophotometric analysis revealed that the insoluble PA levels found in *PtrANR1*-overexpressors were similar to the levels found in the control plants but relatively higher than that in the *PtrANR1-*antisense lines (Fig. 5I). All *PtrANR1*-overexpressing lines except for line 2 showed remarkably increased contents of soluble PAs in their leaves compared with those in the transgenic lines harboring the *PtrANR1-*antisense construct (Fig. 5H). Moreover, all transgenic *35S*:*PtrANR1* lines accumulated higher levels of epicatechin than did the control plants, and no difference (Fig. S4). Quantification of anthocyanin content was achieved by spectrophotometry (530 nm) in transgenic poplar. The *PtrANR1*-overexpressors accumulated much lower levels of anthocyanin when compared with transgenic *PtrANR1-*antisense lines and the empty-vector control (Fig. 5J). Quantitative RT-PCR analysis confirmed reduced *PtrANR1* transcript levels in the antisense lines. These results indicate that constitutive expression of *PtrANR1* in poplar promoted the biosynthesis of PAs but inhibited anthocyanin accumulation.

### *PtrLAR1* Overexpression in Transgenic Poplar Plants

**Figure 6:**
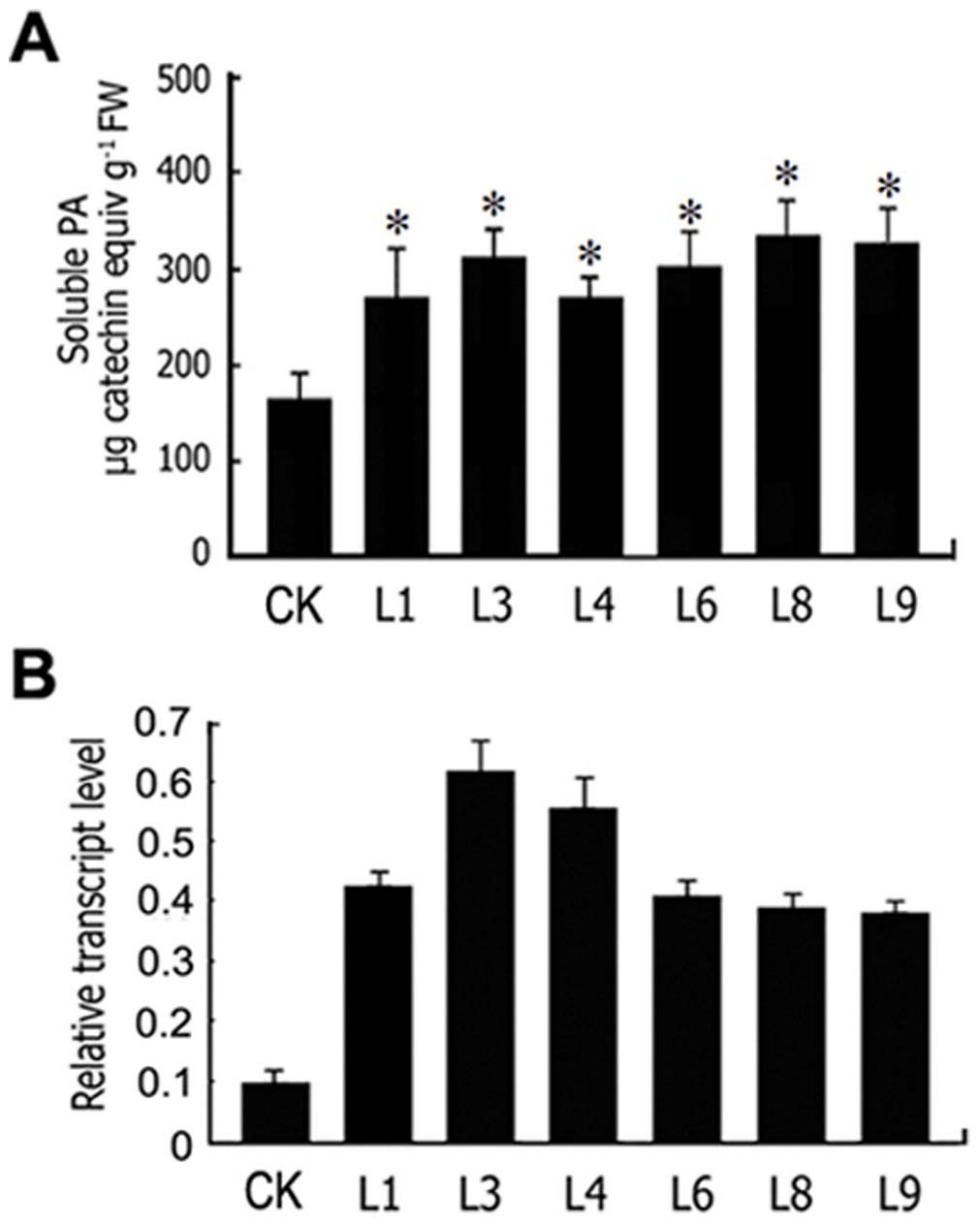
Quantification of PAs and gene expression of *PtrLAR1* in transgenic *35S:PtrLAR1* poplar plants. (A) Soluble PA levels in different transgenic lines. (B) Expression of *PtrLAR1* in different transgenic lines was determined by real-time RT-PCR analysis using *18S* as the housekeeping gene. L1, L3, L4, L6, L8 and L9 refer to independent transgenic *35S:PtrLAR1* lines. CK is the empty-vector control line. Asterisks indicate significant differences using Student’s *t*-test (*P*<0.05). All data is presented as mean of three replicates with error bars indicating ± SD.

The potential function of *PtrLAR1* was investigated in transgenic poplar by expression of a 35S-promoter-driven sense construct. PA accumulation in various tissues of the control and *35S:PtrLAR1* plants were determined. There was a significant increase in the levels of PAs in all transgenic lines compared with the empty-vector control lines (Fig. 6A), consistent with involvement of LAR function in PA biosynthesis. HPLC analysis revealed that transgenic *35S:PtrLAR1* lines accumulated higher levels of catechin and epicatechin, than did the controls (Fig. S5). However, all transgenic lines exhibited a reduction in anthocyanin levels, but flavonols (kaempferol and quercetin) were not significantly changed in the transgenic lines compared with the empty-vector plants (Table 1). Real-time qPCR analysis revealed that overexpression of the *PtrLAR1* transgene was confirmed in the transformed lines (Fig. 6B). These findings indicated that *PtrLAR1* might play important roles in the PA bisosynthesis in poplar.

**Table 1:** Flavonoid contents in the control and transgenic *35S:PtrLAR1* poplar plants^a^.

**Lines**	**Flavonol (ug/g**)		**Proanthocyanidin(µg/g)**		**Anthocyanin** (**µg/g**)
	**Kaempferol**	**Quercetin**	**Catechin**	**Epicatechin**	**Cyanidin**
Control	149.88±0.49	157.51±0.13	10.66±0.66	19.71±0.30	68.48±1.03
*PtrLAR1-*L1	115.69±0.24	151.02±0.40	13.10±0.01	24.13±0.15	57.97±1.54
*PtrLAR1-*L3	115.84±0.56	175.42±0.09	12.37±0.50	25.05±0.01	55.07±1.54
*PtrLAR1-*L4	135.41±0.21	157.10±0.08	12.17±0.01	24.20±0.04	63.41±2.05
*PtrLAR1-*L6	116.85±0.08	151.46±0.06	13.33±0.16	23.66±0.19	28.97±0.51
*PtrLAR1-*L8	N/A	N/A	13.52±0.58	23.69±0.15	N/A
*PtrLAR1-*L9	125.46±0.03	154.16±0.18	13.37±0.15	23.80±0.30	42.02±0.51

a All data correspond to mean values ±SD of three biological replicates. N/A, not available.

## Discussion

Numerous factors like insects and diseases affect the health of perennial poplar plants, resulting in reduced growth. Poplar leaves constitutively accumulate PAs, but their biosynthesis is often up-regulated by biotic and abiotic stresses such as insect herbivory, mechanical wounding as well as pathogen infection [4], [24], [26], [37]. PA accumulation response to wounding and herbivory occurs both locally at the site of damage and systemically in distal leaves [4], indicating that these compounds function in defence against herbivores and pathogens. In the present study, the strong induction of genes encoding the flavonoid pathway enzymes, including *PtrPAL5*, *PtrCHS3*, *PtrCHI, PtrDFR1, PtrANR1* and *PtrLAR1*, was detected in poplar leaves after treatments with wounding, SA and pathogen infection (Fig. 1). The developmentally regulated PA accumulation was found in poplar leaves. Accumulation of PAs began in the youngest leaves (LPI-1) and peaked in mature leaves (LPI-4) (Fig. 4A). This is not consistent with the temporal expression profiles of *PtrANR1, PtrLAR1* and *PtrLAR3* during leaf development (Fig. 4B-D), indicating that PA content constantly increases during leaf development, but the rate of synthesis decreases in old leaves [18].

PAs are important polyphenolic compounds for plant adaptation to the environment, and there lies a considerable interest in their biosynthesis. The PA biosynthetic pathways share common intermediates until leucocyanidin, which may be used by anthocyanidin reductase (ANR) and leucoanthocyanidin reductase (LAR) to produce epicatechin and catechin (precursors of PAs), respectively. ANR has been initially identified in *Arabidopsis*, and it is encoded by the BANYULS (*BAN*) gene [17]. ANR utilizes cyanidin as a substrate, rather than leucocyanidin, which is consistent with the fact that leucoanthocyanidin dioxygenase (LDOX) is essential for PA synthesis in *Arabidopsis* [40]. Ectopic expression of *BAN* in tobacco flower petals and *Arabidopsis* leaves results in loss of anthocyanins and accumulation of condensed tannins, suggesting that there is an interaction between anthocyanidin and PA pathways [17]. Recently, ectopic expression of apple *MdANR* genes in tobacco positively and negatively regulates the biosynthesis of proanthocyanidins (PAs) and anthocyanin, respectively, resulting in white, pale pink-coloured, and white/red variegated flowers [19]. In Our study, *PtrANR1* transcripts are most highly expressed in roots, which are the sites of maximal accumulation of PAs (Fig. 3). Overexpression of *PtrANR1* in poplar resulted in a significant plant-wide increase in PA levels, but it also influences the biosynthesis of anthocyanin by competing with UFGT (UDP-glucose:flavonoid 3-O-glucosyltransferase) activity by which it converts anthocyanidin to anthocyanin (Fig. 5). Antisense down-regulation of *PtrANR1* in poplar reduced the levels of soluble PAs but improved the accumulation of anthocyanin in leaves (Fig. 5). Thus, *PtrANR1* plays an important role in both anthocyanin and PA synthesis in poplar. Our results will provide insights into the interaction between *PtrANR* genes and other genes involved in flavonoid biosynthesis.

To date, *LAR* genes have been isolated from many plant species including tea [41], grape [18], [42], [43]. The LAR activity of correlated with PA accumulation has been clearly characterized [2], [15], [20], [21], [22]. We previously showed that overexpression of *PtrLAR3* in Chinese white poplar (*P. tomentosa* Carr.) resulted in a significant plant-wide increase in PA levels and enhanced fungal resistance in transgenic plants [26]. Here, we have identified *PtrLAR1* and its expression is high in roots (Fig. 3C). Ectopic expression of *PtrLAR1* in poplar induced qualitative and quantitative changes of the proanthocyanidin profiles (Fig. 6), indicating a good correlation of *PtrLAR1* transcript levels with PA accumulation. Based on our studis, both of *PtrLAR1* and *PtrLAR3* contribute to PA accumulation in *Populus*. But obviously, expression level of *PtrLAR3* in *Populus* tissues was higher than that of *PtrLAR1* [26]. Furthermore, overexpression of *PtrLAR3* resulted in more accumulation of PAs in transgenic plants as showed in previous study [26] and Fig. 6. Therefore, these results suggested that *PtrLAR1* and *PtrLAR3* as *LAR* homologs in poplar, are required for PAs accumulation, but *PtrLAR3* play a more significant role than *PtrLAR1* in PA synthesis.

However, PA accumulation was found restricted to some tissues of transgenic plants despite the use of a 35S constitutive promoter. Transgenic tobacco plants overexpressing *MtLAR* showed reduced anthocyanin content, but no catechin or increased levels of PAs were detected either in leaves or in flowers [22], indicating the poor correlation between *MtLAR* expression and PA accumulation. In *Arabidopsis*, PA biosynthetic pathway has been best characterized, but this species accumulates only epicatechin-based starter units and lacks any obvious *LAR* ortholog [40]. In the present work, overexpression of *PtrLAR1* in *Arabidopsis* did not result in an increase in PA levels, although the transgene transcript accumulated at high levels (Data not shown). Thus, *Arabidopsis* does not offer a model system for the genetic study of PA biosynthesis in important crops where parallel catechin- and epicatechin-based pathways contribute to PA biosynthesis [5], [40], [44]. This demonstrates that *Populus* LARs catalyzed the conversion of leucocyanidin to catechin clearly established its role in PA biosynthesis.

In the current model of PA biosynthesis, LAR and ANR provide two separate pathways for the synthesis of the terminal units for PA polymers [3]. It is the absence of evidence whether ANR and LAR provide alternate pathways to PA biosynthesis in different plant tissues or if both branches can be active in some tissues that accumulate high levels of PA. In a previous study, ectopic expression of apple *MdANR* genes in tobacco up-regulated the biosynthesis of proanthocyanidins (PAs) but down-regulated the biosynthesis of anthocyanin. Anthocyanin accumulation was significantly reduced in all tobacco transgenic flowers, while contents of both catechin and epicatechin in transgenic flowers were significantly higher than those in flowers of nontransgenic plants [19]. This finding suggests that the ANR gene may be capable of generating catechin via an unknown route. Here, we found that leaves of all transgenic lines overexpressing *PtrLAR1* accumulated lower anthocyanin than the control plants (Table 1). Interestingly, following HPLC analysis, it was revealed that transgenic lines accumulated higher levels of catechin and epicatechin when compared with the empty-vector control. Moreover, leaves of all transgenic lines produced lower levels of kaempferol than did the control plants (Table 1). It is necessary to understand how the ANR and LAR pathways co-occur and function to contribute to the formation of PAs in the future.

## Supporting Information

Figure S1 **Structure of** ***PtrLAR*** **genes and alignment of deduced amino acid sequences of LAR genes.** (A) Structure of *PtrLAR* genes, black boxes represent exons; lines introns, gray boxes and arrow 5′and 3′ UTRs. The figure is drawn to scale. (B) Alignment of LAR proteins. Sequences are from *L. corniculatus* (ABC71329), *M. truncatula* (XP_003591830), *D. uncinatum* (CAD79341), *D. kaki* (ACI41981), *M. domestica* (AAZ79364), *L. uliginosus* (AAU45392). PtrLAR1 (EEE89746) and PtrLAR3 (EEF06163) are from *P. trichocarpa* (Ptr). Identical amino acids are indicated by white letters on a black background, conservative amino acids by white on a dark gray background, and similar amino acids by black on a light gray background. Asterisks indicate the RFLP, ICCN and THD motifs.
(TIF)

Figure S2 **Structure of** ***PtrANR*** **genes and alignment of deduced amino acid sequences of ANR genes.** (A) Structure of *PtrANR* genes, black boxes represent exons; lines introns, gray boxes and arrow 5′and 3′ UTRs. The figure is drawn to scale. (B) Alignment of ANR proteins. Sequences are from *P. trichocarpa* (XP_002317270*), D. kaki* (BAF56654), *L. corniculatus* (ABC71336), *L. uliginosus* (ABM90632), *M. truncatula* (AAN77735), *M. domestica* (AEL79861), *V. vinifera* (BAD89742). Identical amino acids are indicated by white letters on a black background, conservative amino acids by white on a dark gray background, and similar amino acids by black on a light gray background.
(TIF)

Figure S3 **PCR analysis of transgenic poplar plants.** (A) Genomic DNAs were isolated from kanamycin-resistant plants transformed with the *35S*:*PtrANR1* and *PtrANR1-*antisense vectors. (B) Genomic DNAs were isolated from hygromycin-resistant plants transformed with the *35S*:*PtrLAR1* vector. M, DL2000 DNA Marker; WT, wild-type plants; P, corresponding plasmid DNA (positive control).
(TIF)

Figure S4 **Composition of PAs in transgenic** ***35S:PtrANR1*** **and** ***35S:PtrANR1*** **-antisense (AS) plants.** (A) Quantification of catechin in the empty-vector plants (CK), transgenic *35S:PtrANR1* and *PtrANR1*-antisense plants. (B) Quantification of epicatechin in the control plants (CK) and different transgenic lines. Numbers refer to independent transgenic lines. All data is presented as mean of three replicates with error bars indicating ± SD.
(TIF)

Figure S5 **PA levels in transgenic poplar plants constitutively expressing** ***PtrLAR1*** **.** Accumulation of catechin and epicatechin was analyzed by HPLC analysis. (A) CK is empty-vector control line. (B) PtrLAR1-L4 refers to independent transgenic line L4.
(TIF)
