# Supplementary figures and images for: Isolation and Characterization of cDNAs Encoding Leucoanthocyanidin Reductase and Anthocyanidin Reductase from Populus trichocarpa
Source: PLoS One. 2013 May 31;8(5):e64664. doi: 10.1371/journal.pone.0064664 (PMC3669385; doi:10.1371/journal.pone.0064664)

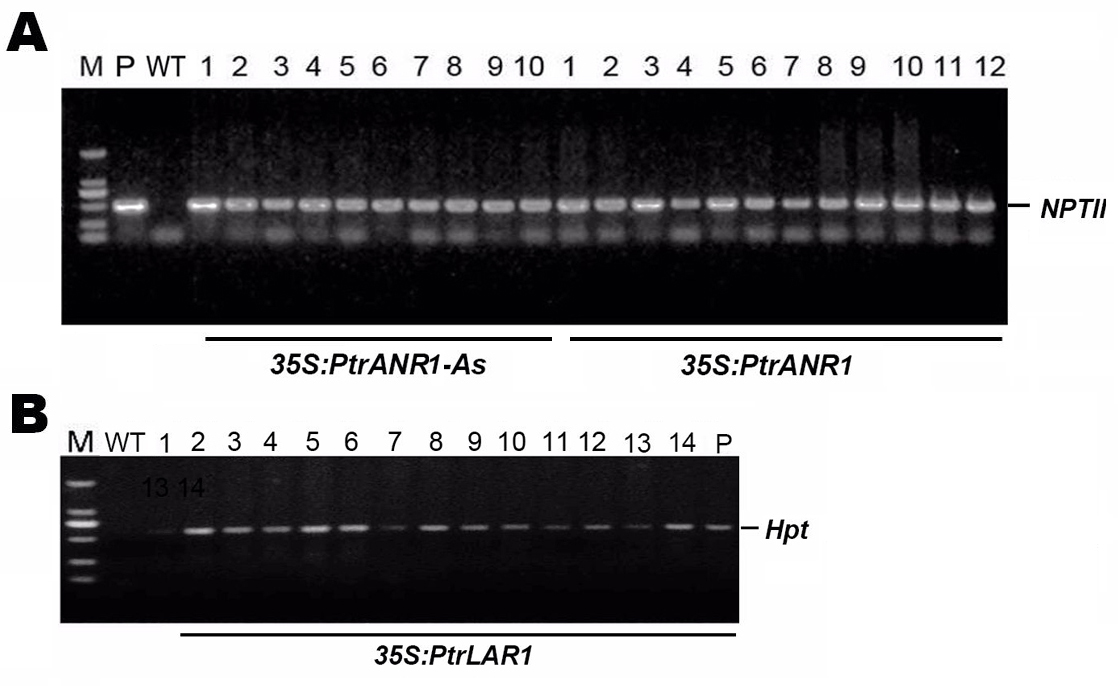

Supplement: Figure S3 — PCR analysis of transgenic poplar plants. (A) Genomic DNAs were isolated from kanamycin-resistant plants transformed with the 35S:PtrANR1 and PtrANR1-antisense vectors. (B) Genomic DNAs were isolated from hygromycin-resistant plants transformed with the 35S:PtrLAR1 vector. M, DL2000 DNA Marker; WT, wild-type plants; P, corresponding plasmid DNA (positive control). (TIF) [file pone.0064664.s003.tif]

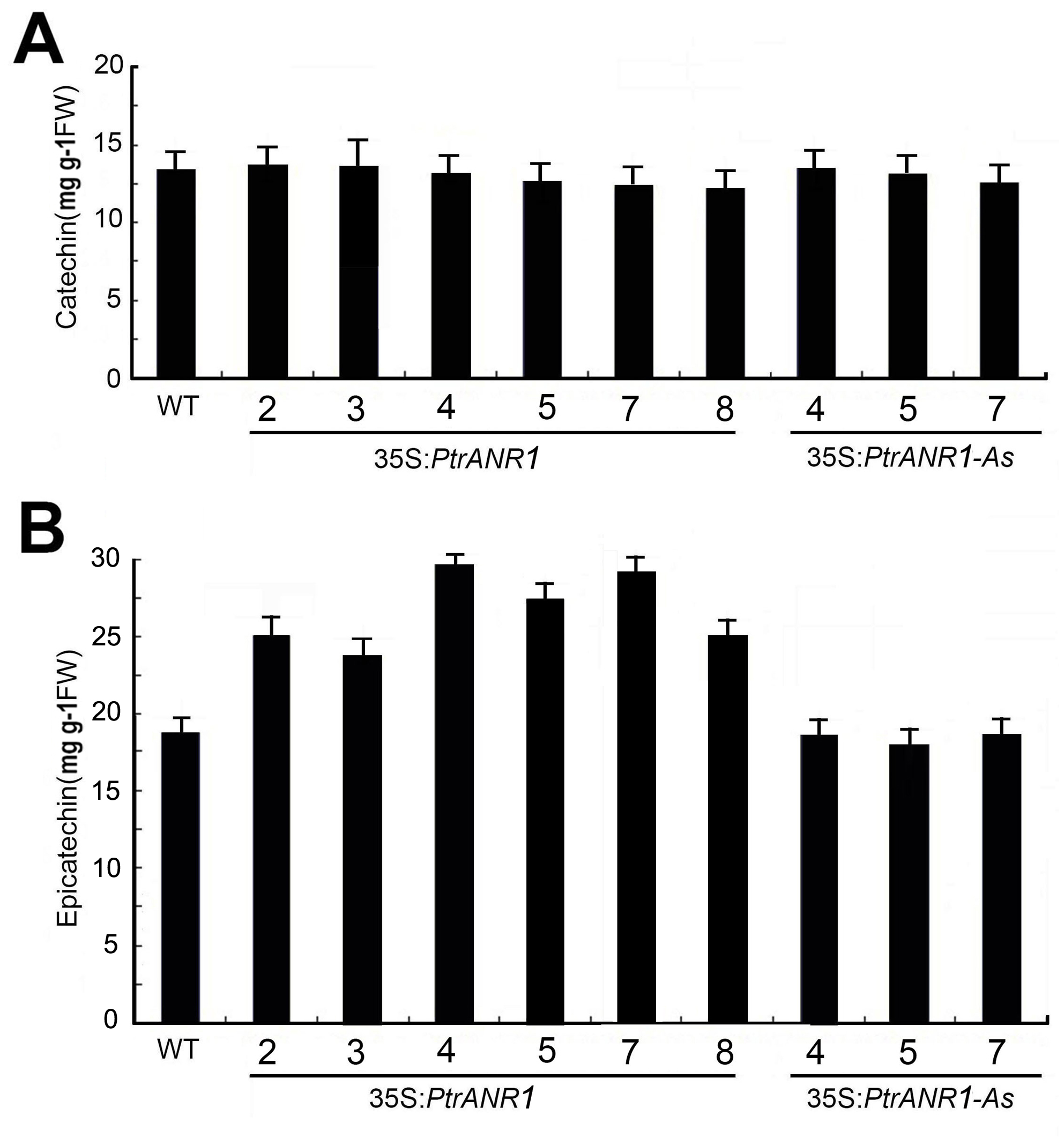

Supplement: Figure S4 — Composition of PAs in transgenic 35S:PtrANR1 and 35S:PtrANR1 -antisense (AS) plants. (A) Quantification of catechin in the empty-vector plants (CK), transgenic 35S:PtrANR1 and PtrANR1-antisense plants. (B) Quantification of epicatechin in the control plants (CK) and different transgenic lines. Numbers refer to independent transgenic lines. All data is presented as mean of three replicates with error bars indicating ± SD. (TIF) [file pone.0064664.s004.tif]

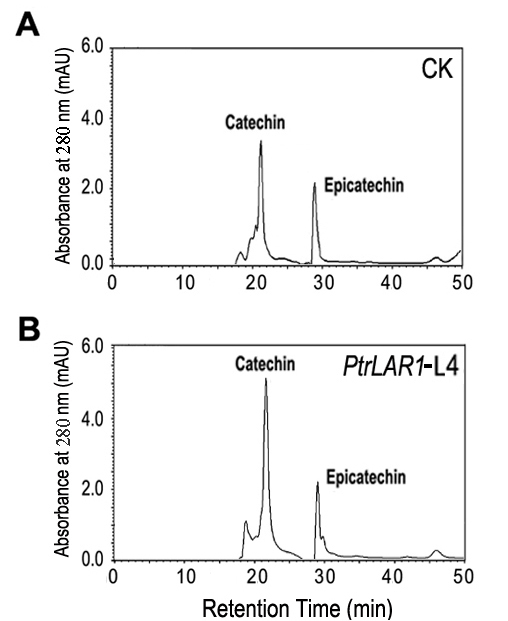

Supplement: Figure S5 — PA levels in transgenic poplar plants constitutively expressing PtrLAR1 . Accumulation of catechin and epicatechin was analyzed by HPLC analysis. (A) CK is empty-vector control line. (B) PtrLAR1-L4 refers to independent transgenic line L4. (TIF) [file pone.0064664.s005.tif]
